# Non-Interventional Prospective Observational Study of Platelet Rich Fibrin as a Therapy Adjunctive in Patients with Medication-Related Osteonecrosis of the Jaw

**DOI:** 10.3390/jcm11030682

**Published:** 2022-01-28

**Authors:** Sebastian Blatt, Maximilian Krüger, Peer W. Kämmerer, Daniel G. E. Thiem, Philipp Matheis, Anne-Katrin Eisenbeiß, Jörg Wiltfang, Bilal Al-Nawas, Hendrik Naujokat

**Affiliations:** 1 Department of Oral and Maxillofacial Surgery, University Medical Center, 55131 Mainz, Germany; Maximilian.Krueger@unimedizin-mainz.de (M.K.); Peer.Kaemmerer@unimedizin-mainz.de (P.W.K.); daniel.thiem@unimedizin-mainz.de (D.G.E.T.); philipp.matheis@unimedizin-mainz.de (P.M.); al-nawas@uni-mainz.de (B.A.-N.); 2 Department of Oral and Maxillofacial Surgery, University Hospital Schleswig-Holstein, Christian-Albrechts-University, 24118 Kiel, Germany; anne-katrin.eisenbeiss@uksh.de (A.-K.E.); Joerg.Wiltfang@uksh.de (J.W.); hendrik.naujokat@uksh.de (H.N.)

**Keywords:** MRONJ, platelet rich fibrin, angiogenesis

## Abstract

Background: Medication-related osteonecrosis (MRONJ) of the jaw is a severe and feared side effect of antiresorptive therapy in the oncological setting. With growing evidence that impaired angiogenesis may represent a key factor in pathogenesis, the aim of this study was to evaluate an autologous platelet concentrate as a possible additive in surgical therapy to optimize vascularization and, subsequently, resolution rates. Material and Methods: A non-interventional, prospective, multicenter study was conducted, and all patients with stage I-III MRONJ, undergoing antiresorptive therapy for an oncological indication, were included. The necrosis was treated surgically without (study arm A) or with (arm B) the addition of an autologous platelet concentrate (platelet-rich fibrin, PRF). Results: After 5, 14, and 42 days postoperative, wound healing (primary outcome: mucosal integrity) as well as downstaging, pain perception, and oral health-related quality of life (secondary outcome) were assessed via clinical evaluation. Among the 52 patients included, primarily with MRONJ stage I and II, the use of PRF as an additive in surgical therapy did not display a significant advantage for wound healing (*p* = 0.302), downstaging (*p* = 0.9), pain reduction (*p* = 0.169), or quality of life (*p* = 0.9). Summary: In conclusion, PRF as an adjunct did not significantly optimize wound healing. Further, no significant changes in terms of downstaging, pain sensation, and oral health-related quality of life were found.

## 1. Introduction

Antiresorptive agents, namely bisphosphonates and receptor activator of nuclear factor kappa-B (RANK) ligand inhibitors, are used to modify bone remodeling and minimize skeletal-related events in osteoporosis, as well as malignancies with bone metastasis such as prostate and breast cancer or multiple myeloma [1,2]. Medication-related osteonecrosis of the jaw (MRONJ), defined as exposed necrotic bone in the oral cavity that persists over 8 weeks, with patients’ history of antiresorptive therapy but without irradiation at the head and neck region, represents a severe and most feared side effect of this therapy with increasing incidence [3,4]. According to the American Association of Oral and Maxillofacial Surgeons (AAOMS), MRONJ can be classified in stage 0-III varying in clinical presentation, inter alia with pain, swelling, (extraoral) fistulae or pathological fractures [5]. Therefore, MRONJ drastically impairs patients’ quality of life, often already suffering from their malignant disease [6]. Prevalence greatly relies on type, duration, dosage, and application method of antiresorptive agents and ranks between 5–12% for high dosage in patients with malignant diseases [7] vs. low dose application in patients with osteoporosis at about 0.01% [8]. Furthermore, there is rising evidence of MRONJ unrelated to bisphosphonates or denosumab—such as immune modulatory medications, tyrosine kinase inhibitors, monoclonal antibodies, mammalian target of rapamycin inhibitors, radiopharmaceuticals, selective estrogen receptor modulators [9] and, as a possible coronavirus disease 2019 therapy option, antiinterleukin-6-receptor (IL-6R) monoclonal antibody [10]. The exact pathophysiological background of the disease is still not clear. Here, five main theories are proposed consisting of inhibition of bone remodeling, infection/inflammation, lack of immune resiliency, altered angiogenesis, soft tissue toxicity [11]. A combined soft- and hard-tissue pathology via direct cytotoxic effects of the antiresorptive agents on fibroblasts, osteoblasts, keratinocytes and endothelial cells is discussed [12]. There is growing evidence that impaired angiogenesis and compromised vascularization may play major role in the pathogenesis of MRONJ [13]. In this context, a recent review identifies the crosstalk between antiresorptive therapy and oral mesenchymal cells and their exosomes as a possible key point of onset of the disease. It was demonstrated that periodontal ligament stem cells (PDLSCs) enhance angiogenesis under inflammatory conditions [14]. Here, in a dose-dependent manner, zoledronic acid significantly impaired viability of this cell population and subsequently induced apoptosis and impaired osteogenic differentiation [15]—and consequently angiogenic potential. A number of controversial aspects exist regarding the management of MRONJ; principally non-surgical and surgical options are administered [16]. In addition to local antiseptics and/or systemic antibiotics, the standard therapy for MRONJ consists of the surgical resection of the necrotic bone and subsequent mechanically stable covering, with well-vascularized soft tissue [17]. Here, long-lasting postoperative mucosal integrity and sufficient wound healing without dehiscence is crucial to further proceed the antiresorptive therapy in oncological indications [7]. However, recurrence/dehiscence rates of up to 60% with increased hospitalization and re-surgery are described in the literature [18]. Overall, in approximately only 30% of the MRONJ cases can disease recurrence be avoided in the long term [19]. Consequently, multiple approaches have been initiated to optimize treatment, such as modification of flap design [17] or intraoperative visualization with fluorescence guided bone surgery [20], but have not yet been translated into clinics. A further possible tactic may represent the adjunctive use of autologous platelet concentrates, such as platelet-rich fibrin (PRF) [21,22]. Since first described by Choukroun et al., PRF as a second-generation autologous platelet concentrate, without addition of any other substrate and producible in only one centrifugation process, is widely used in regenerative medicine and dentistry [23]. The slow and steady release of multiple pro-angiogenic growth factors and cytokines of the fibrin clot enhances proliferation and differentiation of osteoblasts, endothelial cells, chondrocytes, and fibroblasts and may subsequently enhance soft tissue and bony regeneration [24]. Consequently, PRF was shown to be efficient for wound healing as an optimal scaffold for tissue healing processes [25]. Therefore, one may hypothesize that the adjunct of PRF to the surgical treatment of MRONJ has many advantages as follows: foremost, its pro-angiogenic capabilities may overcome the impaired angiogenesis and vascularization and supports soft and hard tissue regeneration. Further, it may be seen as an initial additional layer for a mechanically stable and well-vascularized covering. Lastly, the approach seems very easy to translate into clinical workflow. So far, only insufficient evidence can support the hypothesis and value of PRF in the surgical treatment of MRONJ [26]. Therefore, the aim of this study was to evaluate an autologous platelet concentrate as a possible adjunct in surgical therapy to optimize vascularization and lower wound-healing disorders and disease recurrence when compared to only surgical therapy in terms of wound healing (primary outcome) as well as in downstaging of the disease, pain reduction, and quality of life (secondary outcomes).

## 2. Materials and Methods

### 2.1. Study Design

Due to regulatory reasons, the study was designed to be observational and non-interventional. Patients with stage I–III MRONJ and a history of or current antiresorptive therapy for oncological indications were included, and the surgical treatment adherent to current guidelines was compared to the addition of PRF in the study group.

### 2.2. Setting

The trial was multicenter with the Department of Oral and Maxillofacial Surgery, University Medical Center, Mainz, Germany, and the Department of Oral and Maxillofacial Surgery, UKSH, Christian-Albrechts-University, Kiel, Germany, involved. The local ethics committee approved the following experiments (Ethics committee Chris-tian-Albrechts-University, Kiel, Germany, nr: D 511/19, Landesärztekammer Rhine-land-Palatine, nr: 2019-14723-NIS) in accordance with the ethical standards as laid down in the 1964 Declaration of Helsinki. The study was registered via the German Clinical Trials Register (DRKS, nr: DRKS00019938), and approval was received by the Federal Institute for Vaccines and Biomedicines (Paul-Ehrlich-Institut, nr: NIS535). This study was funded by the “Deutsche Forschungsgemeinschaft” (grant number BL 1701/1-1; NA 1592/1-1)

### 2.3. Participants

**Figure 1:**
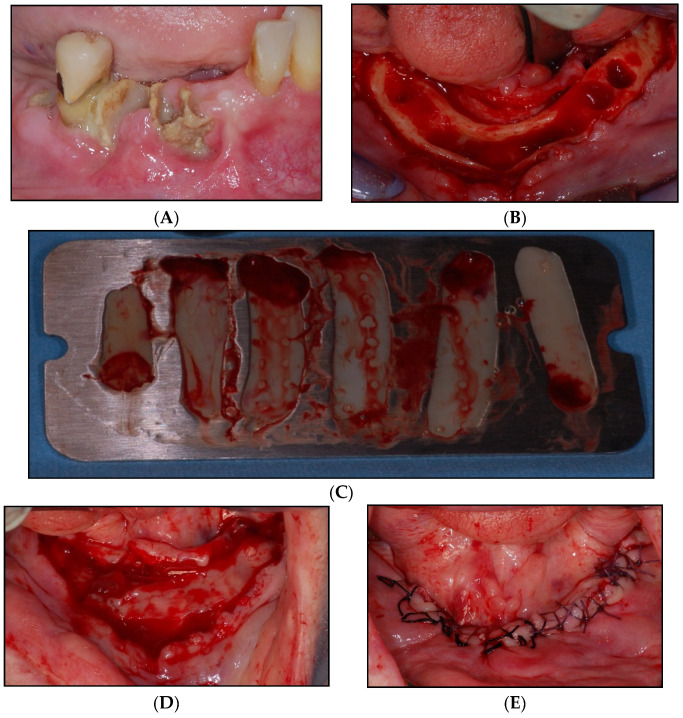
Exemplary surgical procedures in study arm (**B**): (**A**) Preoperative situation with MRONJ stage II of the right mandible with exudation of pus. (**B**) Intraoperative situation after tooth extraction and decortication. (**C**) Pressed PRF. (**D**) PRF covering the decorticated bone. (**E**) Mechanically stable covering with soft tissue.

Patients with stage I-III MRONJ and a history of or current antiresorptive therapy for oncological indications were included. Further inclusion criteria are as follows: patients had to be 18 years or older and able to give consent. All patients gave written informed consent. Patients with a positive history of radiotherapy in the head and neck region, with antiresorptive therapy for an indication other than oncological such as osteoporosis, patients without exposed necrotic bone (MRONJ stage 0), with previous reconstructive surgery with microvascular flaps in the head and neck region, and with metastasis of their malignant disease on the side of osteonecrosis were excluded from the study. Furthermore, pregnant patients and patients already enrolled in other interventional clinical trials as well as patients with hemorrhagic diathesis and/or a thrombocytopenia (platelets below 150,000/µL) were excluded. Before the operation, the size of the necrosis was assessed via cone beam computer tomography. Experienced attending oral and maxillofacial surgeons performed all surgical interventions. The interventions were all conducted under general anesthesia; all patients received perioperative antibiotic therapy with ampicillin/sulbactame 3 g i.v. 1-1-1 (or in case of penicillin allergy: Clindamycin 600 mg p.o. 1-1-1) as well as tube feeding until the 5th postoperative day. Perioperative analgesic therapy was applied individually in adherence with WHO guidelines. The surgeons each decided on their own which study arm a patient should be assigned to. In study arm A, necrotic bone and fistulas (if existing) were resected and a sufficient, vascularized, mechanically stable wound coverage using local soft tissue was performed with absorbable and/or non-absorbable (vicryl and/or silk) single button sutures. In study arm B, patients received the same treatment but additionally received PRF membrane on the decorticated bone before covering. PRF was processed intraoperatively as described in the literature [19]. Briefly, after venous blood collection of 60 mL via special vacutainer systems (A-PRF+; Process for PRF, Nice, France), blood was immediately centrifuged according to the manufacturer’s protocol (1200 rpm for 8 min, relative centrifugal force 177 g at a fixed angle rotor with a radius of 110 mm; Duo centrifuge, Process for PRF, Nice, France). Lastly, a stable PRF membrane was produced though manual press with the PRF-box (Process for PRF, Nice, France) and applied (Figure 1). Patients gave informed consent, inclusion and exclusion criteria were checked, and demographic and baseline characteristics, including medication, were assessed.

### 2.4. Variables, Data Sources/Measurement

The included patients were followed-up postoperatively for six weeks (42 days). After the operation procedure and inclusion of patients in V1 (5th postoperative day), patients in both study arms received perioperative antibiotic therapy and tube feeding until the 5th postoperative day. On that day, before discharge of the patient, clinical visit 2 (V2) was conducted evaluating the following: besides staging via AAOMS criteria, the integrity of the mucosa and wound healing was assessed via the IPR (I = inflammatory, P = proliferative, R = remodeling) Wound Healing Scale as suggested by Hamzani et al. [27]. Briefly, the score is used depending on the wound healing phase. The inflammatory phase is evaluated 35 days postoperative on the basis of bleeding (spontaneously or on palpation), granulation tissue, hematoma, tissue color, incision margins, suppuration, edema, and pain, all measured on a 9-point scale (0–8, a score of 5–8 is considered a successful inflammatory phase). The proliferative phase is evaluated 14 days postoperative on the basis of re-epithelialization, tissue color, scar, suppuration, and pain (measured on a 6-point scale (0–5, whereby a score of 3–5 indicates successful healing)). The remodeling phase is evaluated 6 weeks postoperative on the basis of scar, tissue color, and pain (measured on a 4-point scale (0–3, whereby a score of 2–3 indicates successful healing)). Combined, the IPR scale’s total score ranges from 0 to 16, and 11–16 is considered excellent healing [27]. Additionally, the revised photographic wound assessment tool (PWAT) was used [28]. Here, eight items were evaluated, each measured on a 5-point scale (with 0 indicating the lowest and 5 the highest expression of the respective item) as follows: wound size, depth, necrotic tissue type, total amount of necrotic tissue, granulation tissue type, total amount of granulation tissue, edges, and peri-ulcer skin/mucosa viability [28]. Furthermore, pain assessment was conducted via the visual analogue scale (VAS, 0: no pain, 10: strongest pain). In addition, oral health-related quality of life (OHRQoL) was assessed via German short form of oral health impact profile (OHIP G14) as previously described [29]. Briefly, the occurrence of 14 items related to functional and psychosocial aspects patients experienced as a result of complaints with their teeth, mouth, or dentures were measured on a 5-point scale (with 4 very often and 0 never, total sum: 0–56) [29]. Lastly, possible adverse events were recorded. Clinical visits were scheduled after 14 (V3) and 42 (V4) days postoperative and all mentioned questionnaires (VAS, OHIP G14, IPR, PWAT) were completed at all time points. Sutures were removed at V3 in all cases. At V4, resumption of antiresorptive therapy in oncological indication was assessed (Table 1).

### 2.5. Study Size

Biometrical planning and analysis were performed with support by the Clinical Research Centre Kiel, Germany. For this pilot trial, proper sample size calculation could not be achieved since the standardized effect size was not clear. In accordance with the sample sizes of comparable studies and after retrospective analysis of the clinical files of the respective center (considering inclusion and exclusion criteria and an estimated dropout rate of 10%, approximately 0.8 patients per week were eligible during the funding period), recruiting 25 patients per study arm was targeted. Data was collected at the respective clinical visits and analyzed after the last patient reached the study end (first patient in: 3 July 2020, last patient out: 18 August 2021).

### 2.6. Statistical Analysis

Statistical analysis was conducted with SPSS Statistics for Windows, Version 25.0 (IBM, Ehningen, Germany). Due to the observational character of the study, all evaluated parameters were analyzed exploratively. To check for noteworthy differences between study arms at the respective time points, the two-side Wilcoxon rank sum test and Fisher’s exact test was applied. A *p* value < 0.05 was defined as statistically significant. Boxplots were used for data illustration.

## 3. Results

### 3.1. Participants

**Figure 2:**
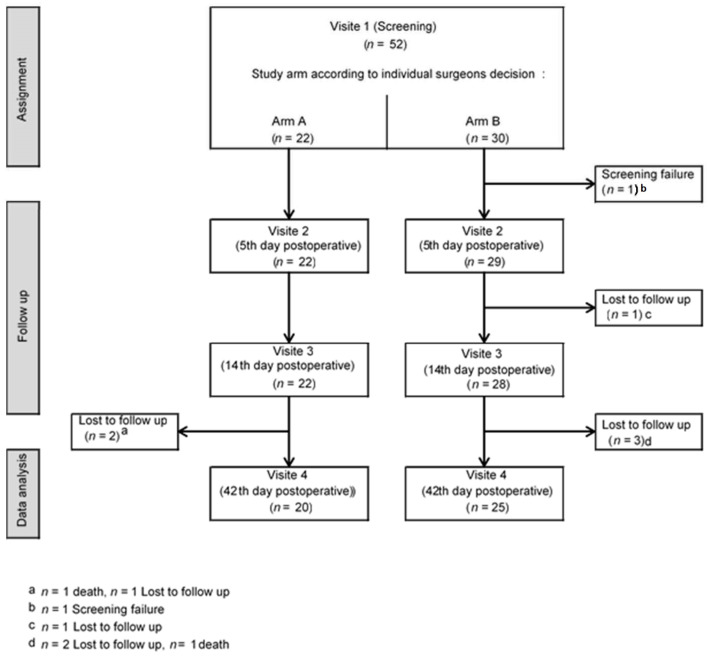
Illustration of patient acquisition and full exclusion details.

Overall, of the 52 patients approached, 7 patients were excluded due to screening failure (*n* = 1) and lost to follow-up (*n* = 6, Figure 2). One patient was lost to follow-up after V2, five patients after V3.

### 3.2. Descriptive Data

The patients’ mean age was 71.5 ± 8.6 years; 27 patients (51.9%) were female. Diabetes mellitus was present in six patients (11.5%), a rheumatic disease in three patients (5.8%), and osteoporosis in 6 patients (11.5%). In 10 patients (19.2%), the risk factor smoking was present, with the majority of these patients smoking 10 cigarettes or fewer a day (*n* = 6, 60%). Mainly because of renal cell carcinoma or non-small cell lung carcinoma, ten patients additionally received immunomodulatory therapy in addition to their antiresorptive intake (19.2%). Breast cancer was the most frequent oncological indication for antiresorptive medication (*n* = 21, 40.4%), followed by prostate cancer (*n* = 12, 23.1%) and multiple myeloma (*n* = 11, 21.2%). As antiresorptive medication, all patients received either Denosumab (XGEVA 120 mg s.c. every four weeks) or bisphosphonates (Zoledronate (Zometa 4 mg/100 mL i.v. every four weeks), Pamidronate (Aredia 90 mg/100 mL i.v. every four weeks)).

The patients mostly showed MRONJ in stage I (*n* = 41, 78.8%), followed by stage II (*n* = 10, 19.2%), and one patient in stage III (*n* = 1.9%). The mean radiological size of the necrosis was 255 ± 1161 mm. In 25 cases (48.1%), the MRONJ had already been treated before, with 12 patients (48%) having had more than one previous oral surgery due to their MRONJ. These previous procedures were performed an average of 10 ± 16.9 months ago.

### 3.3. Outcome Data

**Figure 3:**
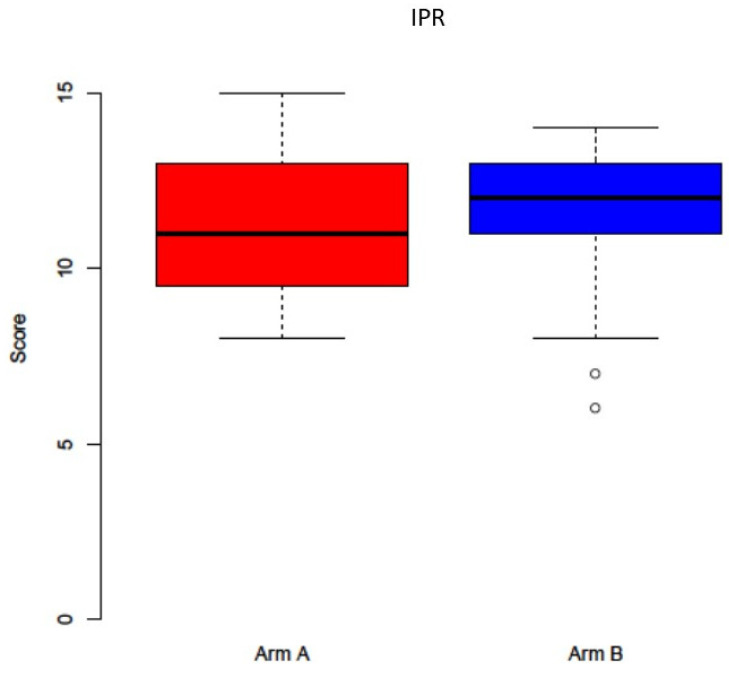
The IPR wound healing score showed no statistical differences between study arm A (−PRF, red boxplot) and B (+PRF, blue boxplot, *p* = 0.302).

**Figure 4:**
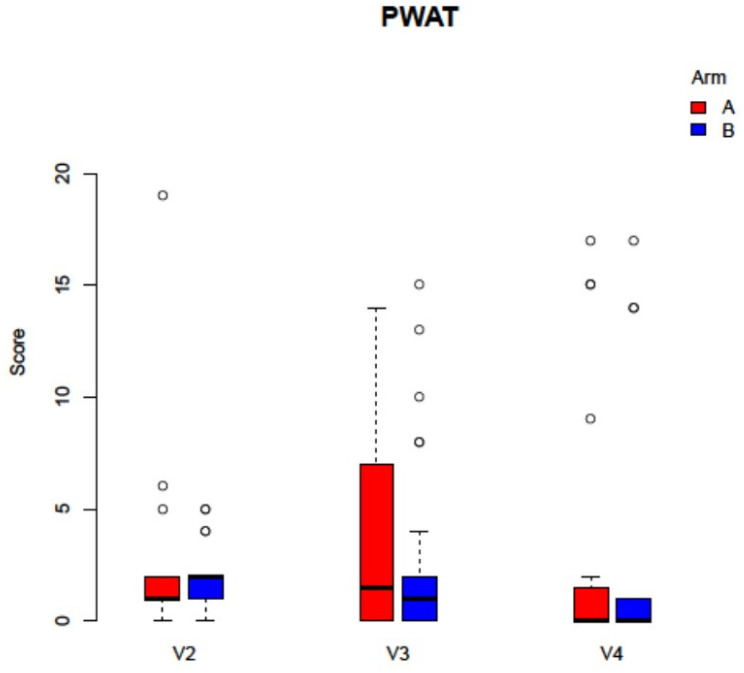
Analysis of the PWAT score did not display any statistically significant differences between the study arms (Arm A: −PRF, Arm B: +PRF, V2 *p* = 0.222, V3 *p* = 0.504, V4 *p* = 0.3017).

During the follow up interval of six weeks, dehiscence and mucosal integrity after MRONJ surgery was observed in sixteen cases (30.76%). There was no statistical difference between the study arms, where eight adverse events occurred in arm A and eight in arm B. The IPR wound healing score showed no statistically significant differences between the two study arms for the overall score (*p* = 0.302, Figure 3). When investigating the different visits, there was also no statistical significance for the respective scores for V2 (*p* = 0.802), V3 (*p* = 0.507), and V4 (*p* = 0.852). Between the single questions of the IPR, There was also no statistically significant differences between the two study arms (each *p* > 0.05). In accordance, the PWAT score did not display any statistically significant differences between the study arms for V2 (*p* = 0.222), V3 (*p* = 0.504), and V4 (*p* = 0.3017), and between the different items (each *p* > 0.05, Figure 4).

### 3.4. Other Results

**Figure 5:**
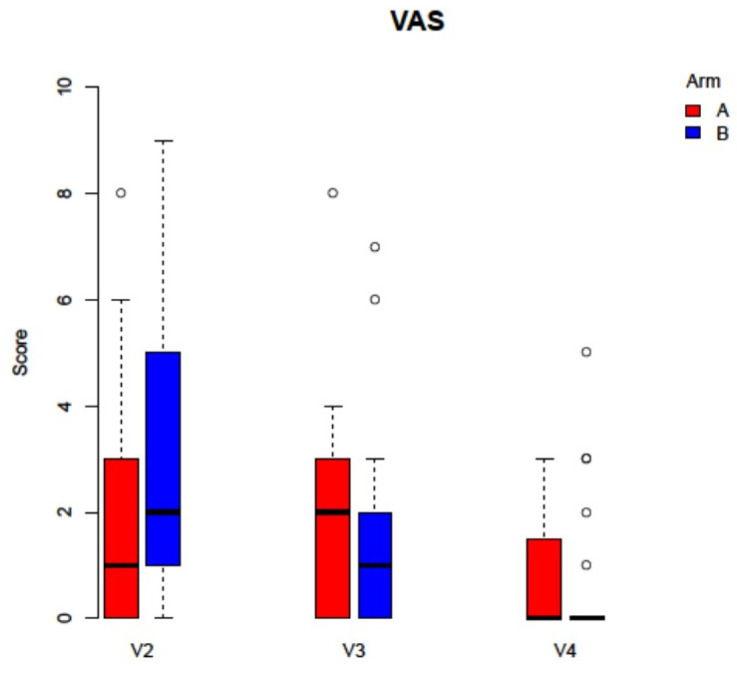
Analysis of the VAS score did not display any statistically significant differences between the study arms (Arm A: −PRF, Arm B: +PRF, V2 *p* = 0.169, V3 *p* = 0.406, V4 *p* = 0.155).

**Figure 6:**
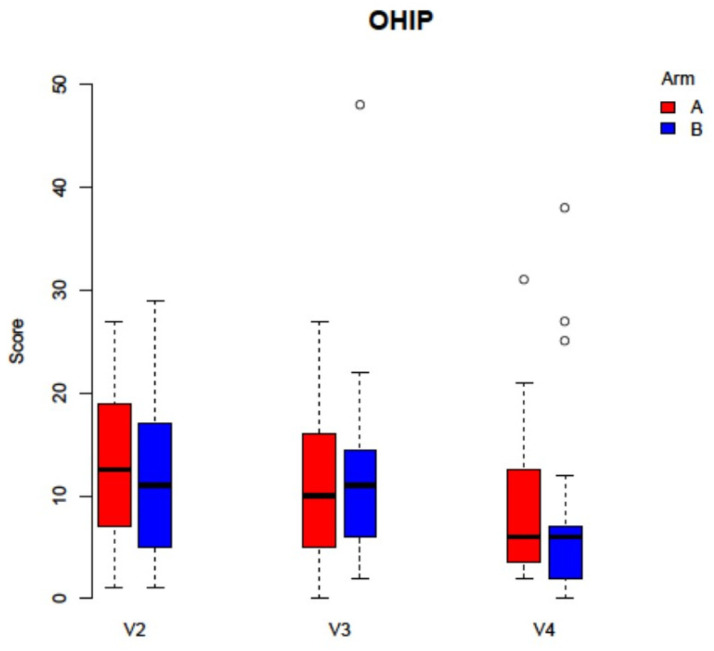
Analysis of the PWAT score did not display any statistical significances between the study arms (Arm A: −PRF, Arm B: +PRF, V2 *p* = 0.222, V3 *p* = 0.504, V4 *p* = 0.3017).

No statistically significant differences in downstaging could be found between the two study arms (V2 *p* = 0.9, V3 *p* = 0.302, V4 *p* = 0.5). For pain sensation, the analysis of VAS did also not demonstrate any statistically significant differences (V2 *p* = 0.169, V3 *p* = 0.406, V4 *p* = 0.155, Figure 5). Concerning OHRQoL, the addition of PRF did not significantly alter answers to the OHIP G14 questionnaire (V2 *p* = 0.965, V2 *p* = 0.746, V3 *p* = 0.154, Figure 6). During follow up time, 12 patients (27.9%) were able to re-start antiresorptive medication after a mean of 30 ± 16.5 days. Four patients (8.9%) could be supplied with dental prosthesis.

## 4. Discussion

In this observational prospective clinical trial, the addition of PRF in the surgical therapy of MRONJ was postoperatively evaluated with respect to wound healing and mucosal integrity, as well as pain sensation, downstaging and oral health-related quality of life. The major result of this study is that the addition of PRF did not statistically influence wound healing nor pain sensation nor the quality of life of the patients analyzed in this cohort. The overall dehiscence rate was 30.76% after surgery during the follow up interval. There were no differences between the study arms. The demonstrated resolution rate is within the range described in the evident literature [30,31]. In a recent review, the pooled proportion of clinical efficacy of surgical clinical efficacy was described even higher at 86%; however, patients with osteoporosis and consequently low dose antiresorptive therapy were also included in this analysis [32].

The exact pathophysiological background of the MRONJ is not fully understood, but it seems to comprise a complex interplay between direct cytotoxic effects on different cell lines [12], suppression of bone remodeling, constant microtraumas, local infection [33] and anti-angiogenic properties [13]. Impaired neovessel formation seems to have a particularly major impact on the pathogenesis of MRONJ [34]. Due to a lack of comprehensive preclinical (large animal studies) and clinical studies, no definitive pathogenesis has been identified yet [35]. The elusive role of the immune response in the development of MRONJ as a key factor in the pathogenesis remains unclear [36]. Here, a study by Paschalidi et al. found that the M1–M2 macrophage polarization status correlates with clinical stage of MRONJ (M2 polarization for early stage, M1 phenotype for advanced stage MRONJ) [37]. This emphasizes the critical role of immunological reactions in the pathogenesis. The full understanding of immunological reactions leading to MRONJ and its involvement in osteogenesis and angiogenesis may represent the missing link to the comprehensive understatement of the disease. These considerations may partly explain the results obtained in this study, where the proven strong pro-angiogenic properties of the PRF, which are known to be strong [38,39,40,41,42], were demonstratively insufficient to significantly optimize soft tissue healing in MRONJ.

As a possible further explanation of the retrieved results, one must reconsider the inter-individual differences between PRF as an autologous platelet concentrate. Besides different procuring protocols, it was shown that membrane size and release of the pro-angiogenic growth factors of the respective autologous platelet concentrate, such as PRP or PRF, highly depend on age and gender [43,44,45].

Nonetheless, the concept of using autologous platelet concentrates such as PRF as a possible adjunct in MRONJ therapy was found to be effective and safe in a recent review. Since MRONJ reportedly has a significant impact on the general and oral QoL especially of cancer patients [46], PRF as an additional surgical treatment option may help to reduce this negative impact. Within a pooled analysis of low to very low quality of evidence (mainly case reports), a success rate over 6 months’ follow-up of 86% was described. In summary, the authors conclude that well-controlled studies are required to make definitive statements [47]. In the review by Govarts et al., the addition of PRF showed 60–100% success for mucosal healing [48]. The presented results in this study are within this range. In their retrospective study, Sánchez-Gellego Albertos et al. found a slightly lower recurrence rate, with approximately 20% after local debridement and application of a first-generation autologous platelet concentrate (platelet-rich plasma, PRP) [49]. So far, no prospective trial evaluates the possible (dis)advantages between the different generations and agents of the respective autologous platelet concentrates. Inchingolo et al. conducted a single study group and found PRF an effective method for the closure of bone exposure in MRONJ patients [50]. This is in accordance with a multitude of case reports that demonstrate PRF to be a valuable adjunct in the surgical management of MRONJ [51,52,53,54].

In this study, only patients with an oncological indication for their antiresorptive therapy were included. In a recent review that summarized the results of two different studies that mainly assessed PRF therapy in osteoporotic patients, PRF achieved full resolution in only approximately 36% of cases [55]. It should be further evaluated in future studies whether the different patient collectives differ in terms of response rate to PRF. A recent review by Fortunato et al. found no differences between surgical treatment alone and application of PRF, but they concluded that current evidence is not sufficient to establish the effectiveness of autologous platelet concentrates [56]. The proposed additional analgesic effect of the PRF [51], which is also described for tooth extraction [57] and insertion of dental implants [58], could not be verified in this study. In this context, a recent study found numb chin syndrome as a further (initial) symptom of MRONJ [59]. Here, autologous platelets may represent a possible scaffold with significant neurological recovery and pain reduction [60]. As a general limitation, one must consider the low to very low quality of evidence of most of the studies found in the current literature for PRF as a possible therapeutical additive in MRONJ surgery [61]. Considering this, so far management strategies such as early vs. late surgery vs. conservative treatment of MRONJ seem clinically more important for patients’ overall outcome than potential additives such as autologous platelet concentrates. For prevention and additional treatment, a drug holiday from the antiresorptive therapy has been discussed and evaluated as effective in a MRONJ large animal model with minipigs under zoledronate therapy [62]. In addition to prevention strategies, especially timing and extent of surgical interventions are still controversially discussed. Here, decision making is guided by the extent of the disease, reflected by the actual staging system [63]. However, the AAOMS staging is often termed as insufficient since the anatomical/radiological extent of the disease is not mapped [47]. There is increasing evidence that early surgical intervention may lead to higher healing rates, compared to (long-lasting) conservative treatment, and may therefore be reconsidered also in early MRONJ stages [64,65]. Since no accurate staging system reflects the extension of MRONJ bone disease, the European Task force on MRONJ recommends early surgical intervention on localized disease that may prevent progression and the need for subsequent extensive surgery [16]. A study by Guidice et al. found that surgically treated patients with MRONJ stage I (and II) showed “benefits in outcomes such as mucosal integrity and lesion downstaging, improvement in quality of life, and faster reuptake of medication therapy especially for oncologic patients” [66]. This is in accordance with the findings of this study where patients with MRONJ stage I were treated surgically with a favorable outcome, independent of the study arm. It seems that conservative therapy of stage I diseases may be powerful in the preservation of the symptomatic status quo, whereas early surgical intervention, as proposed in this study, favors complete removal of the necrosis and long-term mucosal healing in order to attain the retrieval of the physiological condition [64]. Since the majority of the patients in this study had early stage MRONJ, the conclusions drawn should be applied for this patient collective. In this context, a recent study by Tenore et al. found a 100% healing rate for PRF as a surgical adjunctive in stage I-II MRONJ, but the PRF in the study group was in addition to photobiomodulation and comparisons were not made to PRF alone [67]. In summary, treatment protocols are complex and need to be adapted to the individual patient, leading to an individual decision [64].

This study suffers from some drawbacks as follows: Although a control group was integrated, the major limitation of the presented study is the preliminary and observational study character with a small sample size. As a possible confounder, there is a numerical discrepancy between the included MRONJ stages of the respective patients in this analysis. Here, the majority of the patients presented with early (stage I) disease, consequently the results should be interpreted with caution for higher stages. Furthermore, the considerable short follow-up is another drawback of this study. Within this work, only patients with oncological indication of their antiresorptive therapy were analyzed, and there was no subanalysis between patients receiving bisphosphonates and RANK ligand inhibitors. Lastly, the fact that approximately half of the patients included in this study already received MRONJ treatment before inclusion in this study may represent another confounder of the presented results.

## 5. Conclusions

In conclusion, given the stated limitations, this observational study did not find PRF as a therapy additive effective in significantly optimizing wound healing, in comparison to the regular surgical approach in adherence to existing guidelines. Further, no significant changes in terms of downstaging, pain sensation and oral health related quality of life could be demonstrated. Future randomized control trials are much in need to validate the role of autologous platelet concentrates in MRONJ therapy.

## Figures and Tables

**Table 1:** Layout and timeline of postoperative procedure and visits (VAS: visual analog scale, OHIP G14: German short form of oral health impact profile, PWAT: revised photographic wound assessment tool, X = investigation done).

	Visit 1 (Screening)	Visit 2 (5th Day Post op.)	Visit 3 (14th Day Post op)	Visit 4 (42nd Day Post op.)
Informed consent	X			
In-/exclusion criteria	X	X		
Demographic/baseline characteristics	X			
Staging according to AAOMS	X	X	X	X
VAS		X	X	X
OHIP G14		X	X	X
IPR		X	X	X
PWAT		X	X	X
discharge		X		
Suture removal			X	
resumption of antiresorptive therapy				X
Adverse events		X	X	X
Medication	X	X	X	X

## Data Availability

The data used to support the findings of this study are included within the article.
